# A stumbling block or a stepping stone?

**DOI:** 10.1007/s12471-016-0818-6

**Published:** 2016-02-15

**Authors:** F. M. Zimmermann, L. R. Dekker

**Affiliations:** Department of Cardiology, Catharina Hospital Eindhoven, Eindhoven, The Netherlands

**Answer**

Fabry disease is a lysosomal storage disorder caused by deficient activity of α-galactosidase A (α-Gal A) resulting in lysosomal accumulation of globotriaosylceramide. The glycolipid accumulation occurs in several organs including the heart, resulting in left ventricular hypertrophy and valvular heart disease. Typical ECG abnormalities include left ventricular hypertrophy, ST-T changes, pathological Q waves in the absence of myocardial infarction, and first-degree atrioventricular (AV) block [1].

We present a case of possible delta waves on the ECG. The differential diagnosis includes pre-excitation or ‘pseudo delta waves’ caused by abnormal depolarisation as a result of the Fabry disease. Second-degree AV block (Mobitz II) is present on the emergency room ECG. Since conduction over the accessory pathway is independent of AV conduction, in case of true pre-excitation and AV block the atrial activity would be conducted via the accessory pathway to the ventricle. However, one can clearly see that at the moment of AV block, accessory pathway conduction is absent. Therefore the ‘delta wave’ cannot be caused by pre-excitation, but is the result of abnormal depolarization, a so-called pseudo delta wave. The patient was treated with a DDDR pacemaker and is doing well 2 months after this episode.

Although more common in some other lysosomal storage disorders, pre-excitation is not commonly seen in Fabry disease. High-grade AV block is also rare and is possibly related to globotriaosylceramide deposition in the conduction system [1, 2].

## Conclusion

Mobitz II AV block in a patient with Fabry disease and pseudo delta waves.
